# Editorial: Epidemiology and molecular epidemiology of childhood and adolescent cancers

**DOI:** 10.3389/fpubh.2023.1177707

**Published:** 2023-03-21

**Authors:** Logan G. Spector, Kevin Y. Urayama, Juan Manuel Mejia-Arangure

**Affiliations:** ^1^Division of Epidemiology and Clinical Research, Department of Pediatrics, University of Minnesota, Minneapolis, MN, United States; ^2^Department of Social Medicine, National Center for Child Health and Development, Tokyo, Japan; ^3^Graduate School of Public Health, St. Luke's International University, Tokyo, Japan; ^4^Facultad de Medicina, Universidad Nacional Autonoma de Mexico, Mexico, Mexico; ^5^Genomica del Cancer, Instituto Nacional de Medicina Genomica, Mexico, Mexico

**Keywords:** acute lymphoblastic leukemia, cancer in children and adolescents, epidemiology, molecular epidemiology, cancer

During the month of February, the international day marking the fight against childhood cancer is commemorated (1). There are many achievements of note about its treatment and improvements in understanding the pathology of these diseases (2). Issues related to prevention are less certain for most types of childhood cancer. Currently it is difficult to try to prove the causality of an environmental factor in the etiology of cancer in childhood and adolescence.

Environmental factors interact with the individual's genetic factors to develop cancer and this interaction could be more marked in childhood. There are scenarios that will allow us to get closer to the causality of childhood cancer, molecular epidemiology has been providing us with a theoretical and analytical framework to identify interactions, gene-environment, gene-gene, etc. (3).

This Research Topic included several manuscripts which give an overview of how childhood cancer behaves in various regions—including China (Zhu et al.) and Mexico City (Flores-Lujano et al.)—as well as a look at the behavior of cancers in children under 5 years of age on a global scale (Ren et al.). While some cancers have been decreasing in recent years in children under 5 years of age, including leukemia, Mexico City has shown a constant increase in this type of cancer in recent years.

This Research Topic also addressed the importance of looking for germline mutations in adolescent and childhood tumors (Shahani and Marcotte). On the other hand, the contrast is also shown that in some countries the identification of gene rearrangements is not in routine clinical use in some cancers, such as myeloid leukemia in children in Mexico City (Sepúlveda-Robles et al.).

This Research Topic highlighted the role of the Hispanic population in understanding some pathologies such as acute lymphoblastic leukemia and hepatoblastoma. While in leukemia the Hispanic population has the highest frequency, data from Texas shows that this population is the most predisposed to developing metastasis in hepatoblastoma (Espinoza et al.).

The issue of cancer in adolescents is a topic that needs to be addressed more frequently. Since the young population is more and directly exposed to various environmental and behavioral factors that may be related to cancer at this stage of life, such as cigarettes, alcoholic beverages, radio frequencies generated by the use of cell phones, tablets, viral infections and even occupational exposures. The participation of genetic factors is different, considering the age at which they appear, while gestational risk factors may have less relevance than in cancers with early childhood onset. Cancers such as osteosarcoma, gonadal tumors, and myeloid leukemia begin to appear more frequently in these age groups.

The issue of cancer in adolescents has been less studied, when these pathologies in this age group could allow us to better understand how cancer originates, since sometimes adolescents have a great predisposition to cancer and yet do not develop it during their early childhood, but present cancer until reaching adolescence. An example of this is osteosarcoma, where the participation of genetic alterations associated with cancers that occur very early, such as retinoblastoma, has been seen, and yet for some reason these children do not develop that cancer, nor do they develop cancer so early, until they reach adolescence. On the other hand, it is also interesting that the exposure to some environmental factors that are generally associated with cancers in adult life, may be related to cancers in adolescence, such is the case of the previous use of alkylating agents (4, 5). This age group should be studied in greater depth.

There will always be an advantage to publishing an article in a Research Topic, as it allows interested researchers to view a concentration of interesting and recent articles on the subject you wish to address in one place. The Frontiers publishing house has been characterized by promoting international groups to form editorial groups that can address issues of common interest. The three editors of this Research Topic are members of the CLIC, which is an international strategy to try to understand the origin of cancer in children and adolescents and that this allows us to meet the challenge of epidemiology and public health, to be able to prevent these diseases.

## Author contributions

All authors listed have made a substantial, direct, and intellectual contribution to the work and approved it for publication.
